# Striatal fibrinogen extravasation and vascular degeneration correlate with motor dysfunction in an aging mouse model of Alzheimer’s disease

**DOI:** 10.3389/fnagi.2023.1064178

**Published:** 2023-03-09

**Authors:** Hanna E. Berk-Rauch, Arnab Choudhury, Allison T. Richards, Pradeep K. Singh, Zu-Lin Chen, Erin H. Norris, Sidney Strickland, Hyung Jin Ahn

**Affiliations:** ^1^Patricia and John Rosenwald Laboratory of Neurobiology and Genetics, The Rockefeller University, New York, NY, United States; ^2^Department of Pharmacology, Physiology and Neurosciences, Rutgers-New Jersey Medical School, Newark, NJ, United States; ^3^Brain Health Institute, Rutgers University, Piscataway, NJ, United States

**Keywords:** fibrinogen extravasation, vascular degeneration, striatum, motor dysfunction, Alzheimer’s disease

## Abstract

**Introduction:** Alzheimer’s Disease (AD) patients exhibit signs of motor dysfunction, including gait, locomotion, and balance deficits. Changes in motor function often precede other symptoms of AD as well as correlate with increased severity and mortality. Despite the frequent occurrence of motor dysfunction in AD patients, little is known about the mechanisms by which this behavior is altered.

**Methods and Results:** In the present study, we investigated the relationship between cerebrovascular impairment and motor dysfunction in a mouse model of AD (Tg6799). We found an age-dependent increase of extravasated fibrinogen deposits in the cortex and striatum of AD mice. Interestingly, there was significantly decreased cerebrovascular density in the striatum of the 15-month-old as compared to 7-month-old AD mice. We also found significant demyelination and axonal damage in the striatum of aged AD mice. We analyzed striatum-related motor function and anxiety levels of AD mice at both ages and found that aged AD mice exhibited significant impairment of motor function but not in the younger AD mice.

**Discussion:** Our finding suggests an enticing correlation between extravasated fibrinogen, cerebrovascular damage of the striatum, and motor dysfunction in an AD mouse model, suggesting a possible mechanism underlying motor dysfunction in AD.

## 1. Introduction

Accumulating evidence indicates a critical role for cerebrovascular dysfunction in AD development and progression. Epidemiological studies indicate there is a strong correlation between risk of AD development and vascular disorders such as atherosclerosis, hypertension, and hypercholesterolemia (de la Torre, 2002; Humpel, 2011). Cerebral blood flow is decreased in AD and chronic hypoperfusion may play a role in occurrence and pathology of AD (de la Torre, 2006; Austin et al., 2011; Mazza et al., 2011). Morphologically, microvessels are misshapen and their number is reduced in AD patient brains, resulting in an increased presence of string vessels, connective tissue remnants of no longer functional vessels (Brown, 2010; Iadecola, 2013). Several other alterations of the cerebrovascular unit occur in AD patient brains, such as disruption of the blood–brain barrier (BBB) (Iadecola, 2013).

While the exact mechanism underlying vascular dysfunction in Alzheimer’s disease is unclear, strong evidence suggests that the interaction of amyloid β (Aβ) with fibrinogen, a large glycoprotein that is the primary component of blood clots may, in part, be responsible. Aβ binds directly to fibrinogen and alters thrombin-induced fibrin clotting, as well as clot lysis by plasmin (Ahn et al., 2010; Singh et al., 2018). AD mouse models also exhibited altered thrombosis and fibrinolysis, and reduction of fibrinogen, additionally disruption of the Aβ/fibrinogen interaction ameliorates AD pathology and cognitive deficits in AD mice (Ahn et al., 2014; Singh et al., 2018). Furthermore, elevated levels of extravasated fibrin(ogen) accumulate in the brains of Alzheimer’s patients, as well as in AD mice, where it contributes to increased vascular injury, inflammation, and short-term memory loss (Cortes-Canteli et al., 2010, 2015; Cajamarca et al., 2020). Excessive fibrinogen increases endothelial wall permeability by binding to and altering tight junctions, as well as interacting with several immune cells resulting in activation of proinflammatory pathways (Tyagi et al., 2008; Patibandla et al., 2009; Davalos and Akassoglou, 2012).

Although progressive cognitive decline is the major symptom of AD, 10–40% of AD patients exhibit signs of motor dysfunction, including gait, locomotion and balance deficits early in disease progression (O'Keeffe et al., 1996; Goldman et al., 1999; Scarmeas et al., 2004; Buchman and Bennett, 2011; Albers et al., 2015) while the majority of patients experience severe motor impairment during later stages of disease (Allen et al., 2003; Kumar et al., 2021). Furthermore, changes in motor function may proceed other symptoms of AD as well as correlate with increased severity and mortality (Albers et al., 2015). Despite the frequent occurrence of motor dysfunction in AD patients, little is known about the mechanisms by which this behavior is affected. Motor and balance functionality is controlled by a complex neuronal network, of which the striatum plays a central, organizing role. The striatum receives input from disparate regions of the cerebral cortex where striatal neurons then process and project this information to the motor cortices *via* the thalamus, forming the cortico-striatal-thalamic circuit (Foster et al., 2021). Thus, neurodegeneration in this region, as is seen in Parkinson’s disease (PD), initiates progressive motor dysfunction, such as the tremors observed in these patients. Interestingly, Huntington’s disease studies suggest that the GABAeric projection neurons which make up 95% of the neuronal population of the striatum are uniquely vulnerable to excitotoxic injury as they lack certain neurotrophic protections compared to other neuronal cell types (Rikani et al., 2014). Damage and death of this neuronal population leads to progressive loss of motor function resulting in symptoms such as impaired gait, muscle rigidity and loss of fine motor skills (Bunner and Rebec, 2016). The potentially deleterious effects of AD progression on the striatum are not well elucidated, however multiple MRI studies have noted a significant decrease in the caudate volume of AD patients as compared to non-demented controls (Madsen et al., 2010; Stein et al., 2011).

While human and animal model studies have shown motor and balance dysfunction in AD (Ewers et al., 2006; Albers et al., 2015; O’Leary et al., 2018), the specific brain regions and the molecular basis for these behavioral phenotypes have not been elucidated. In several other neurological diseases, such as stroke and vascular parkinsonism (VP), cerebrovascular impairment underlies motor dysfunction during the progression of the diseases (Park, 2016; Li et al., 2021). Severe effects on motor function are seen in stroke patients whose infarctions occur in the basal ganglia, evidence of a relationship between vascular dysfunction and alter motor capacities (Hatem et al., 2016). Together these observations raise the possibility that deficits in the vascularity may underlie some of the motor dysfunction exhibited by AD patients and in AD mouse models. In this study, we found that fibrinogen-associated vascular damage and axonal demyelination occur in the same region of the aging AD mouse brain, the striatum and appear to have some region specificity, as occurrence is higher in the striatum than the cortex. Furthermore, manifestation is during a time period of declined motor function in these mice providing an intriguing correlation between impaired motor function and blood vessel abnormalities in the AD.

## 2. Materials and methods

### 2.1. Animals

Tg6799 mice (The Jackson Laboratory) are double transgenic mice for APP/Presenilin 1 that express five early onset familial AD mutations on a mixed background C57BL/6 × SJL (Oakley et al., 2006). Tg6799 male mice aged 7 months and 15 months were used for all behavioral and immunohistochemical analysis. Wild-type (WT) littermates were used in all experiments as controls. The genotype of all the mice used in this study was reconfirmed by taking tail tissue on the day of sacrifice. All mice were maintained on a 12-h light/dark cycle and given *ad-lib* access to irradiated mouse chow and water for the duration of the experiment. All experiments were done according to policies on the care and use of laboratory animals of the Ethical Guidelines for Treatment of Laboratory Animals of the NIH. Relevant protocols were approved by the Rockefeller and Rutgers Institutional Animal Care and Use Committee (IACUC).

### 2.2. Immunohistochemistry for vascular and fibrinogen staining

Mice were saline/heparin-perfused, and humanly sacrificed. Brains were harvested, washed in PBS, and snap frozen with dry ice in cryomolds. Following freezing, 20um coronal brain sections were produced on a Leica CM 1900 Cryostat. These sections were fixed with 50% methanol and 50% acetone prior to immunohistochemistry staining. For fibrinogen and endothelial cell staining, brain sections were incubated with FITC-conjugated anti-fibrinogen antibody (Dako; 1/1,000 dilution) and anti-CD31 antibody (BD Biosciences; 1/40 dilution) overnight. To detect the CD31 staining an Alexa Fluor 555 goat anti-rat secondary antibody was used in 3% normal goat serum for 1 h at room temperature (Invitrogen; 1/1,000 dilution). Sections were treated with 0.3% Sudan Black B (sigma) in 70 EtOH for 1 min at room temperature with slight agitation to reduce auto-fluorescence.

### 2.3. Immunohistochemistry for Aβ deposits and microglia

To detect activated microglia staining and Aβ deposits, brain sections were incubated with anti-CD11b antibody (DSHB;1/20 dilution), and an Alexa Flour 488-conjugated Monoclonal anti-Aβ antibody 6E10 (Covance;1/1,000 dilution overnight). For detection of CD11b an secondary antibody conjugated to Alexa Fluor 555 (Invitrogen; 1/1,000 dilution) was used in 3% normal goat serum for 1 h at room temperature. Sections were treated with 0.3% Sudan Black B (sigma) in 70 EtOH for 1 min at room temperature with slight agitation to reduce auto-fluorescence.

### 2.4. Myelin and axon staining

To investigate the integrity of myelin structure in the white matter of the striatum, Luxol Fast blue staining (Abcam) was performed according to manufacturer’s instruction. Briefly, 20 μm frozen coronal sections were fixed and incubated with Luxol Fast Blue solution for 24 h at room temp. Sections were submerged with lithium carbonate solution and further in ethanol to differentiate. Then sections were counter stained with Cresyl Echt Violet for 2–5 min and dehydrated with absolute alcohol following which they were mounted on coverslips. Immunostaining for myelin was performed with an antibody against Myelin Basic Protein (MBP; 78,896, Cell Signaling Technology). Axonal staining was performed using an antibody against neurofilament heavy chain (AB5539, Millipore) in the cortex and striatum separately or with MBP. The immunohistochemical images were quantified in Image J (NIH) software.

### 2.5. Image analysis

After immunohistochemistry, brain sections were analyzed with a confocal microscope (Inverted DMI 6000; Leica) equipped with HyD detectors at room temperature using HCX PL APO CS 20 × (NA 0.7) objective. To minimize biased stereology, confocal images of whole coronal brain sections were collected by tile scan using Leica Application Suite Advanced Fluorescence software. Each set of stained sections was processed under identical gain and laser power setting as well as brightness and contrast settings. The following workflow was used in image J to analyze the confocal images. The blood vessel density in each brain region was determined by measuring the area of CD31 staining as a percentage of the total area of each region and the number of infiltrated fibrinogen deposits were determined by counting fibrin(ogen) positive/CD31 negative staining in each brain region. Similarly, the total area of Aβ deposits or activated microglia was analyzed by measuring the Aβ staining or CD11b (microglia) staining as a percentage of total area of specific brain region. In all these analyses, an average of the area or count number from 4 to 6 different sections from each mouse were determined (*n* = 5 mice per group) and the researcher was blind to the genotype of each mouse. Coronal brain sections were selected from Bregma coordinates 1.54 to –1.82 mm and they are evenly distributed within the area. These brain sections included striatum, thalamus, hippocampus, and the bulk of the cortex, including somatosensory and motor regions.

### 2.6. Behavioral analysis

All behavioral experiments were performed and analyzed with a researcher blinded to genotype and treatment. Mice were handled and allowed to acclimate to the testing room for 10 min per day for at least 5 days prior to testing. All experiments were conducted with lux values that were uniform.

#### 2.6.1. Open field test

Animals were exposed to a square arena that measured 45 × 45 cm. All animals were given 10 min to freely explore the area. The distance traveled, time spent in center, and overall path direction were recorded automatically with Ethovision software (Noldus, United States).

#### 2.6.2. Elevated plus maze test

Animals were given 5 min to freely explore an elevated maze with four arms (30 cm long and 5 cm wide). Two arms were surrounded by opaque walls (closed arms; with 20 cm walls) while the other two arms termed “open” lacked any enclosure on the walkway. The entire maze was elevated to 35 cm off the ground. Trials were 5 min long for each mouse and were recorded automatically with Ethovision software. Time spent exploring each arm area was measured and the percentage of time in open arms was expressed as percentage of total time spent in maze.

#### 2.6.3. Rotarod test

The accelerating rotarod task was used to measure motor coordination and balance of Tg6799 mice. Each 10-min trial began with a 30-s acclimation period at 4 rpm followed by gradual acceleration to a maximum of 40 rpm over the next 9.5 min. Performance was measured as the latency for an animal to fall off from the rod. Three rotarod trials per day were performed over three consecutive days and the interval between trials in a day was 30 min.

### 2.7. Statistical analysis

All numerical values presented in graphs are mean ± SEM and analyzed using GraphPad Prism Software. Statistical significance of most experiments was determined using Student’s *t* test or two-way ANOVA and *post hoc* pairwise *t*-test with the Bonferroni correction. Comparison of training curves from rotarod test was analyzed using two-way ANOVA with repeated measure and Bonferroni *post hoc* test.

## 3. Results

### 3.1. Increased fibrin(ogen) extravasation and vascular degeneration in the striatum of AD mouse model

Aβ binds to fibrinogen and alters fibrin clot formation and lysis (Ahn et al., 2010; Cortes-Canteli et al., 2010). Furthermore, delayed fibrin clearance and an increased in fibrin(ogen) extravasation have been associated with blood vessel occlusion, neuroinflammation and cognitive dysfunction in transgenic AD mouse models (Cortes-Canteli et al., 2010; Ahn et al., 2014). In prior studies we and others observed that fibrin(ogen) extravasation significantly increases in the cerebral cortex and induces spine elimination and cognitive impairment through microglial activation in Tg6799 mouse, a well characterized familial Alzheimer’s disease model (Ahn et al., 2014; Merlini et al., 2019). Thus, we further analyzed age-dependent fibrin(ogen) extravasation in several brain areas of Tg6799 mice including cerebral cortex, striatum, hippocampus, and thalamus at 7 and 15 months of age to determine the effect of brain region and animal age on fibrin(ogen) extravasation.

**Figure 1:**
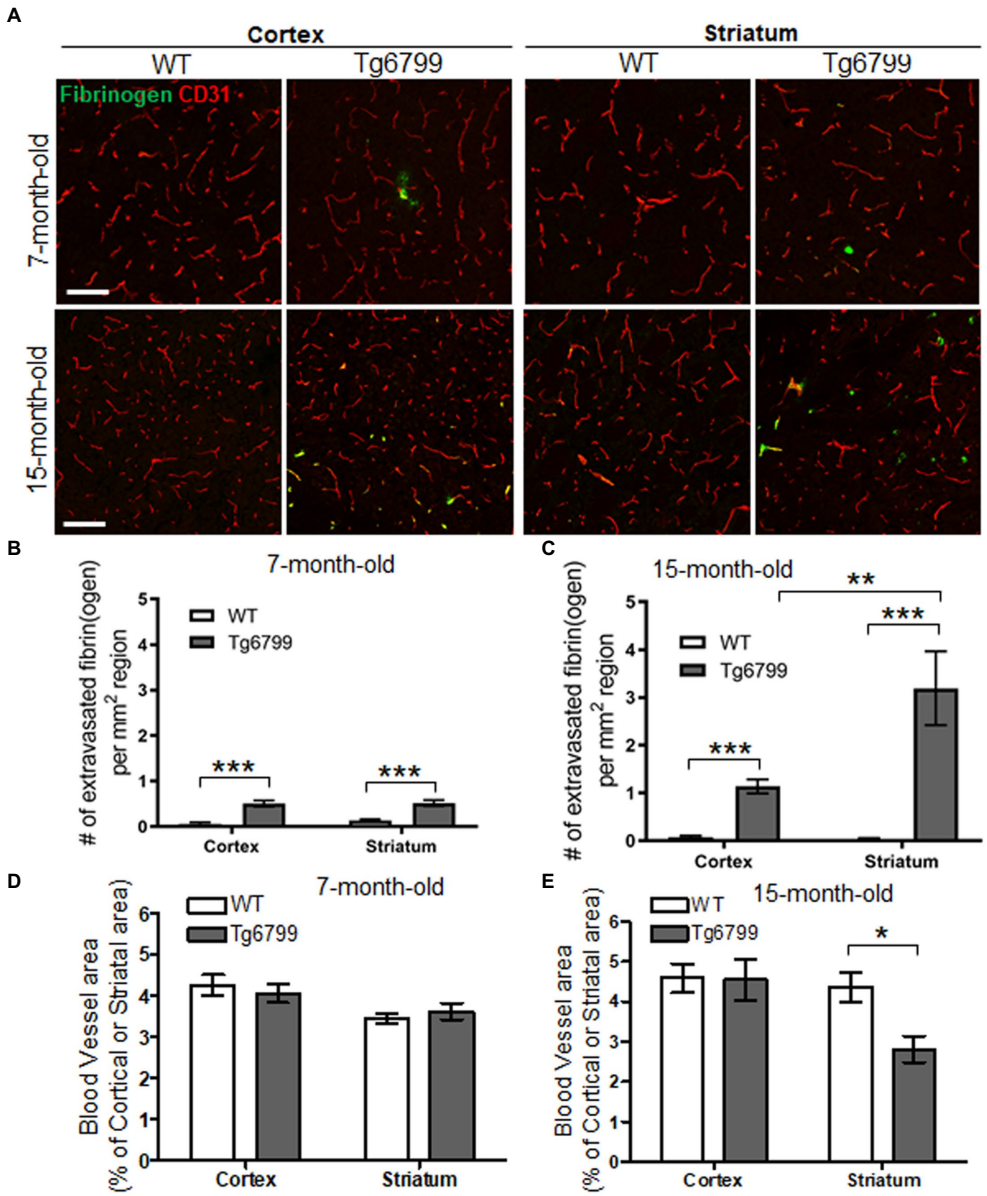
Extravasated fibrinogen and blood vessel density in 7- and 15-months Alzheimer’s disease (AD) mouse model. **(A)** Immunohistochemistry for the blood vessel marker CD31 (red) and fibrinogen (green) was performed on coronal brain sections of 7- and 15-months Tg6799 AD and Wild-type (WT) mice (Scale bar, 50 μm). **(B,C)** Number of extravasated fibrinogen was measured in the stratum and cortex at 7- and 15-months Tg6799 AD and WT mice. **(D,E)** The blood vessel area as a percentage of total brain region (i.e., stratum and cortex) was measured at 7- and 15-month age cohort. All numerical values presented in graphs are mean ± SEM. Statistical significance was determined using two-way ANOVA with *post hoc* pairwise *t*-test was applied by correcting the *p*-values with the Bonferroni procedure (^*^p < 0.05; ^**^p < 0.01; ^***^p < 0.001; *n* = 5 per group).

To assess extravasated fibrin(ogen) deposits in Tg6799 mice, we performed immunohistochemistry for fibrinogen in conjunction with CD31, an endothelial cell marker. We counted fibrin(ogen) deposits at sites where there was a complete lack of co-localization with CD31 as well as at the sites where CD31 was in close proximity with infiltrated fibrinogen, as both suggests that a given deposit is extravasated (Figure 1A). We found that there was significant increase of fibrin(ogen) extravasation in the cerebral cortex and the striatum of the AD mouse brain at both ages compared to wild type (WT) littermates and found an age-dependent increase of extravasated fibrin(ogen) deposits in both regions (Figures 1A–C). Even though we could not find region-specific increase of fibrin(ogen) extravasation in the mice of 7-month cohort between cortex and striatum (Figure 1B), the number of extravasated fibrin(ogen) deposits in the striatum of the 15-month cohort was three times higher than that in the cerebral cortex of the same cohort (Figure 1C). In the hippocampus, fibrin(ogen) extravasation was significantly increased in 15-months-old AD mice, but not in 7-months-old AD mice (Supplementary Figures 1A,B). There was no significant increase of fibrin(ogen) extravasation in both ages of AD mice in thalamus (Supplementary Figures 1A,B). Interestingly, there was profound age-dependent increase (more than 7-time higher) of extravasated fibrin(ogen) in the striatum of the 15-month-old Tg6799 mice compared to 7-month-old Tg6799 mice, while age-dependent increase of extravasated fibrin(ogen) in other brain regions such as cortex and thalamus was minimal.

Extravasated fibrin(ogen) deposits are a morphological signature for fibrinoid necrosis and excessive fibrin(ogen) results in endothelial cell damage, shearing of blood vessel walls and loss blood vessel integrity (Paul et al., 2007; Rosenblum, 2008; Tyagi et al., 2008; Patibandla et al., 2009). We investigated whether increased fibrin(ogen) extravasation is related to vascular degeneration in the cerebral cortex and striatum by assessing the overall blood vessel area in each region. As an indication of blood vessel density, we immunostained for CD31 in both the AD and WT cohorts, at the 7- and 15-month age points and determine the percentage of CD31 positive signal from the total region area (Figure 1A). While we did not observe any differences between genotypes or brain regions for CD31 positive blood vessel area in 7-month cohort (Figure 1D), we found that there was a significant decrease in vascular density in the striatum of the 15-month-old AD mice as compared to their WT counterparts (Figure 1E). The cellular density in the same region was not altered in AD and WT mice assessed by nuclear staining using DAPI (Supplementary Figures 2A,B). This vascular degeneration was restricted to the striatum, as the blood vessel area in the cortex was unaltered between AD and WT mice. This age specific vascular degeneration in the striatum correlates with the pattern of extravasated fibrin(ogen). Interestingly, due to extensive vascular degeneration in the striatum, fibrinogen staining is often observed in the absence of endothelium staining and does not appear to colocalize with blood vessels. These findings suggest an association between increased vascular degeneration and fibrin(ogen) extravasation as they are both observed with high frequency in the striatum.

### 3.2. Increased level of Aβ plaque and microglial activation is associated with striatal vascular degeneration

**Figure 2:**
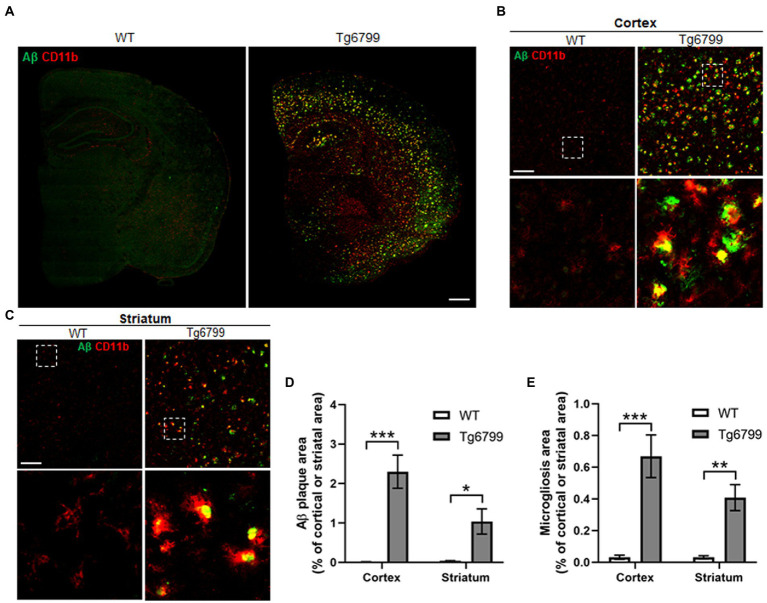
Amyloid β (Aβ) deposition and microgliosis in 15-month old mice. Aβ plaque load and microglial activation were compared between Tg6799 AD and WT mice cohorts at 15-months by immunohistochemistry. Tissue sections were co-stained with the antibody 6E10 (green), specific for human Aβ and an antibody against the microglia marker CD11b (red) to assess plaques and inflammation in the stratum and cortex. **(A)** Lower magnification representative stitched coronal brain hemispheres of WT and Tg6799 shown with 6E10/ CD11b staining distribution in cortex and striatum at 15-month timepoint. (Scale bar, 500 μm). **(B,C)** Representative higher magnification images of Aβ plaque and CD11b positive microglia shown in cortex and striatum of WT and Tg6799 mice (Scale bar, 100 μm). The dotted box indicates magnified area showing association between activated microglial cells and Aβ plaque in cortex and striatum and represented in the lower panel. **(D)** Aβ deposited area was measured as percentage of total cortical and striatal area. **(E)** Microgliosis as a measure of activated microglia area was quantified as percentage of total cortex and striatum area. All numerical values presented in graphs are mean ± SEM. Statistical significance was determined using two-way ANOVA and *post hoc* pairwise *t*-test was applied by correction the *p*-values with the Bonferroni procedure (^*^p < 0.05; ^**^p < 0.01; ^***^p < 0.001; *n* = 5 per group).

The defining feature of Alzheimer’s disease is an increased level of Aβ deposits and microgliosis throughout the brain of afflicted individuals (Luber-Narod and Rogers, 1988; Dickson and Vickers, 2001; Serrano-Pozo et al., 2011). The increased level of Aβ leads to the increase fibrin(ogen) deposits, as well as microglial activation, resulting in neuroinflammation and in turn the downstream pathology seen in Alzheimer’s disease patients (Serrano-Pozo et al., 2011; Cortes-Canteli et al., 2012; Hansen et al., 2018). One possibility is that the increased extravasated fibrin(ogen) in the striatum may result from an increased level of Aβ deposition. To test this possibility, we measured the area of Aβ deposits present in the striatum and cerebral cortex of the 15-month cohort. We found markedly high levels of Aβ deposits in both the cerebral cortex and striatum of AD mice as compared to WT (Figures 2A–D), though the level of Aβ deposits in the striatum was less than that in the cerebral cortex, suggesting that there is not a linear association between Aβ deposits and increased extravasated fibrin(ogen) in the striatum (Figures 2A–D).

Often degenerating blood vessels are due to inflammation of the area surrounding them, which in turn will recruit microglia or macrophage, as well as other immune defenses to the site (Miao et al., 2005; Bell and Zlokovic, 2009). We measured the levels of activated microglia or macrophage, by immunostaining against CD11b, as a means of assessing local neuroinflammation in the striatum and cerebral cortex of the 15-month cohort. Many CD11b positive activated microglial cells were clustered around or adjacent to the Aβ plaque (Figures 2B,C). We found significantly increased levels of activated microglia or macrophage in 15-month AD mice, in both the striatum and cortex (Figure 2E). Thus, these results suggest that Aβ deposition and microgliosis are associated with increased levels of extravasated fibrin(ogen) and vascular degeneration in the striatum of the 15-month AD mice.

### 3.3. Demyelination and axonal damage in the striatum of aged AD mice

**Figure 3:**
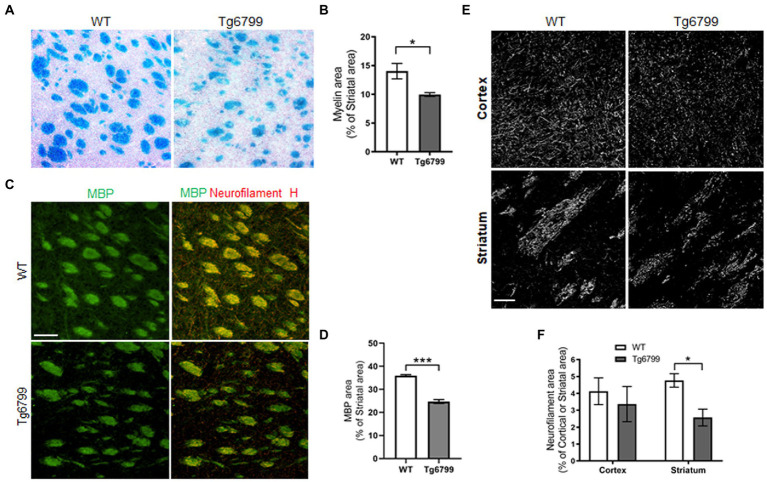
Demyelination and axonal damage in striatum of aged AD model. **(A)** Histological staining using Luxol fast blue stain was used to visualize myelin in the striatum of 15-months Tg6799 AD and WT mice, and myelinated area was analyzed. **(B)** Myelinated area as percentage of total striatum region area was measured in 15-month age cohort. **(C)** Representative images showing striatal Myelin Basic Protein (MBP) immunoreactivity separately and colocalization with neurofilament heavy chain in WT and Tg6799 mice at 15-month age (Scale bar, 100 μm) **(D)** MBP area was quantified as percentage of total striatal area. **(E,F)** Immunohistochemical analysis for axonal damage was visualized and qualified using anti-neurofilament heavy chain antibody in the cortex and striatum of 15-months Tg6799 and WT mice (Scale bar, 50 μm). Data were analyzed by Student’s test or two-way ANOVA and *post hoc* pairwise *t*-test was applied by correction the *p*-values with the Bonferroni procedure and shown as average ± SEM. (^*^p < 0.05; *n* = 4–5 per group).

Myelin is the lipid-rich glial cell plasma membrane component which covers the neural axons in the form of myelin sheaths, and which plays a key role in transmitting electrical signal along neural circuits (Nave and Werner, 2014). Demyelination of axons occur during aging and underlies several neurological conditions in both the central nervous system and spinal cord (Bartzokis, 2004; Love, 2006). In aged brain, demyelination in the white matter of the ventral striatum affects cognitive performance and correlates with cortical microvascular lesions in dementia patients (Kovari et al., 2004; Steiger et al., 2016). In the striatum of 15-month mice, we assessed myelination with the Luxol fast blue staining and determined the percentage of myelinated tissue area as a percentage of total striatal area. By this method, we found a significant decrease in myelin-stained area in striatal white matter of Tg6799 as compared to WT littermates (Figures 3A,B). Striatal demyelination was further confirmed using MBP immunostaining along with neurofilament heavy chain, a major structural component of the axonal cytoskeleton, where we found significant reduction in MBP stained striosome and matrix area in 15-month old AD mice compared to WT mice (Figures 3C,D; Supplementary Figure 3). MBP and neurofilament colocalized signal were also reduced in Tg6799 mice (Figure 3C) indicating potential axonal demyelination in the striatum (Supplementary Figure 3). Previous studies showed that in AD brain, white matter abnormalities such as demyelination were associated both with vascular dysfunction and degenerative axonal loss (McAleese et al., 2021). To assess whether there is axonal loss in addition to the demyelination observed, we stained for neurofilament heavy chain separately in the cortex and striatum of 15-month old AD mice cohort. As expected, we found a significant reduction of axonal neurofilament staining in the striatum of 15-month-old Tg6799 group compared to WT control, which appeared to be region specific as no significant difference was seen in the cortex of the same mice (Figures 3E,F). Taken together, these results suggest that demyelination and axonal damage occur in close proximity to fibrinogen extravasation and vascular degeneration in the striatum of AD mice.

### 3.4. Aging AD mice exhibit decreased motor function

The observation of striatum-specific vascular alterations in the aging AD mouse model, raises the possibility that these mice may also exhibit striatum-related behavioral changes, such as motor function deficits and altered levels of anxiety (Peters et al., 2016; Tewari et al., 2016). To assess this possibility, we performed a series of well-established behavioral assays. Using the open field task, we examined spontaneous locomotor activity of Tg6799 mice by measuring the total distance traveled and additionally determined the ratio of time spent in the center versus total time in the field, an indicator of basal anxiety. We found that there was no statistically significant difference in the distance travelled or the percentage time spent in the center of the field for either genotype or age (Supplementary Figures 4A,B), suggesting there are no gross changes in spontaneous locomotor and basal anxiety at both ages of Tg6799 mice.

**Figure 4:**
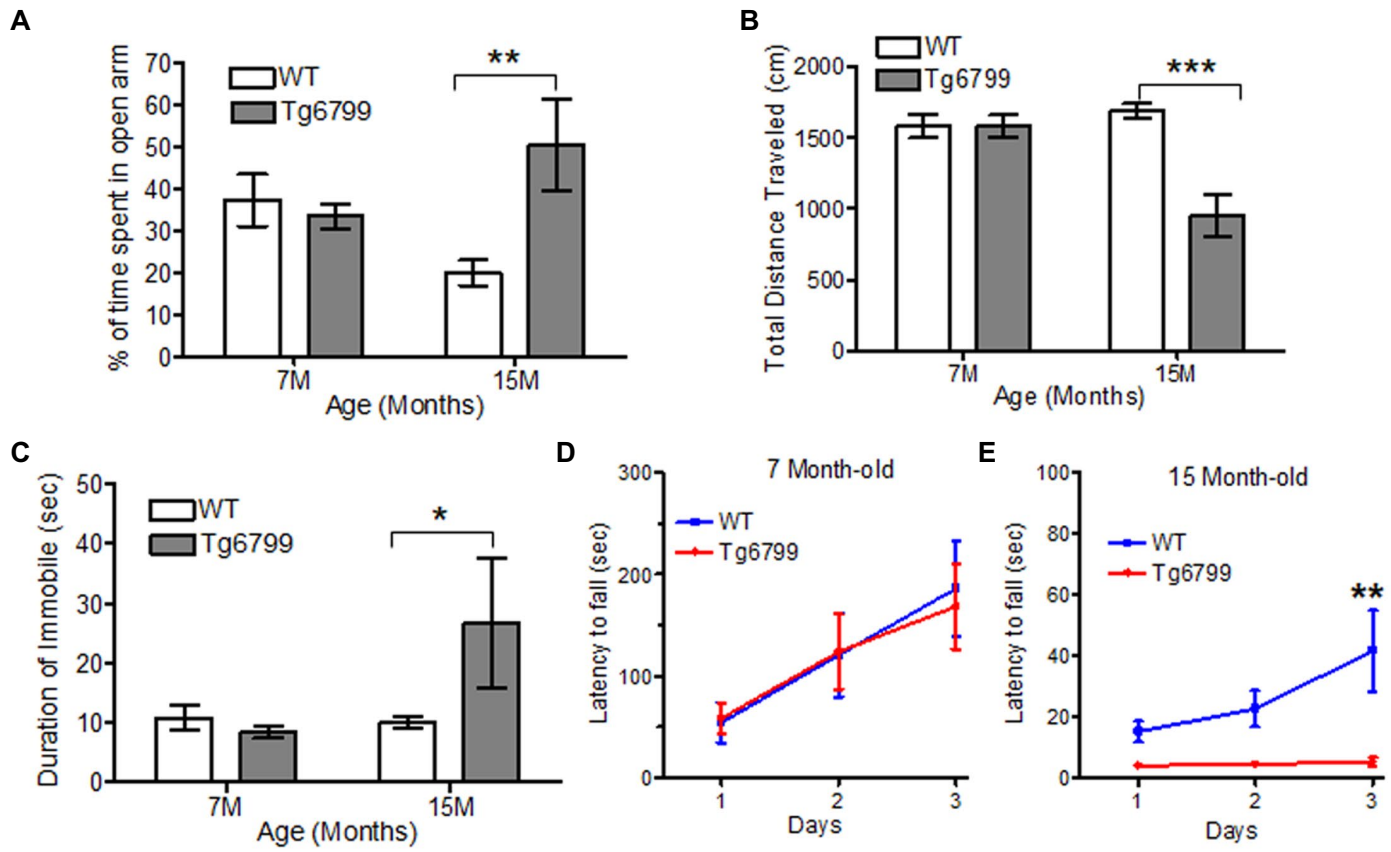
Altered motor function and anxiety behavior in aged AD mice model. **(A–C)** Anxiety and motor behavior was measured in Tg6799 AD and their WT littermate at 7- and 15-month age using Elevated Plus Maze. The amount of time spent by the animal in open arm **(A)**, total distance travelled **(B)** and percent time immobile was measured **(C)**. Balance and motor function in Tg6799 AD and WT controls at 7-month **(D)** and 15-month **(E)** age were performed using rotarod behavioral test. The latency of fall from the rod was measured between AD and WT mice for three consecutive days (*n* = 5–7 per group). Data were analyzed by two-way ANOVA with *post hoc* multiple comparison using Bonferroni procedure and shown as mean ± SEM (^*^p < 0.05; ^**^p < 0.01; ^***^p < 0.001; *n* = 6–10 per group).

To further probe locomotive dysfunction, as well as anxiety, we used the elevated plus maze test. While this assay is thought to, primarily, determine anxiety levels of the mice, it also requires a higher level of dexterity than the open field test, because the mice need to move through narrow arms (5 cm width). We found a significant increase in the amount of time the 15-month-old Tg6799 mice spent in the open arms compared to WT (Figure 4A). This result suggests a general reduction in anxiety in the 15-month AD mice, which is in accordance with other studies of these mice (Jawhar et al., 2012; Schneider et al., 2014). We also noted that the total distance travelled was significantly decreased in the 15-month-old Tg6799 mice (Figure 4B) and these mice spent a significantly increased amount of time without any movements as compared to their WT control (Figure 4C), raising the possibility that the increased time in the open arms may, in part, be due to motor dysfunction. This result appeared contradictory to our findings in the open field test, in which there were no differences between the two genotypes at either age. Therefore, we hypothesize that the tight angles in the elevated plus maze may require higher levels of fine motor coordination from the animals as compared to the open field test.

To test our hypothesis, we examined the motor coordination and balance of the Tg6799 mice using rotarod. While the previous behavioral assays provided indirect evidence of motor deficits, the rotarod is a more straightforward means of assessing motor coordination, balance, and endurance. When challenged with the rotarod behavioral assay, we observed no difference between the genotypes at the 7- and 11-month aged cohort (Figure 4D; Supplementary Figure 4C), but the 15-month Tg6799 mice exhibited a significant decrease in their latency to fall time as compared to their WT control (Figure 4E), indicating a loss of motor and balance function over time in the Tg6799 mice. Altogether, our behavior analysis suggests that vascular degeneration, fibrin(ogen) extravasation, and demyelination in the striatum may contribute to abnormal anxiety and motor dysfunction in an aging Alzheimer’s disease mouse model.

## 4. Discussion

Several lines of evidence indicate a role for cerebrovascular deficits in Alzheimer’s disease development and progression, perhaps due in part to the interaction between Aβ and fibrinogen. In this study we present evidence suggesting a novel linkage between cerebrovascular dysfunction and locomotion deficits in AD. We find a region-specific effect on the striatum during the progression of AD with decreased cerebrovascular density and elevated levels of extravasated fibrinogen as well as demyelination and axonal damage which correlates with an onset of motor dysfunction.

While it seems unexpected that the striatum would be specifically affected in the aging AD mouse model, there are morphological characteristics of this brain region that may in part explain this observation. The striatum is particularly prone to cerebral small vessel disease and high blood pressure correlates with decreased striatum volume (de Jong et al., 2014). The microvessels lenticulostriate arteries, the main type of vascularity in this brain region, have small diameters which may make them less adaptive to increased blood pressure than larger vessels and thus more susceptible to damage. Small blood vessels are particularly prone to fibrinogen-induced injury as a result of damages from changes in blood viscosity and vessel reactivity (Lominadze et al., 2010). For example, over 40 % of strokes and vessel obstruction have been linked to severe obstructions originating in the vasculature of the basal ganglia including the dorsal and ventral striatum (Park, 2016). Furthermore, Aβ induced altered fibrin deposits may resist degradation, and increased fibrin deposits along the vessels may induce endothelial cell degeneration *via* increased inflammation or occlusion of capillaries (Brown, 2010; Cortes-Canteli et al., 2010).

Our current study focuses on investigating a relationship between extravasated fibrinogen, cerebrovascular damage of the striatum, and motor dysfunction in AD. Even though the current study does not directly validate the role of fibrinogen in AD pathogenesis, many previously published studies showed direct evidence. Specifically, both our group and others have published several studies delineating the mechanism of fibrinogen in the pathogenesis of AD and cerebral amyloid angiopathy (CAA). It has been shown that fibrin clots formed in the presence of Aβ are structurally altered and resistant to degradation (Cortes-Canteli et al., 2010, 2015). In the case of CAA, known disease-causing mutations increase the interaction between fibrinogen and Aβ, resulting in severely altered fibrin clot structure and resistance to fibrinolysis as well as increased fibrin(ogen)/Aβ co-deposition in the brain of patients (Cajamarca et al., 2020). Moreover, fibrinogen extravasation, CAA, and gliosis in the cortex were significantly reduced by the inhibition of fibrinogen-Aβ interaction and reduction of fibrin levels using anti-coagulant rescued AD pathology and improved cognitive function in mouse models of AD (Ahn et al., 2014; Cortes-Canteli et al., 2019). Several other publications also reported that fibrinogen induces neuroinflammation *via* microglial activation or astrocyte scar formation, both of which are linked to cerebrovascular damage and cognitive decline in AD (Schachtrup et al., 2010; Merlini et al., 2019). Additionally, stereotactic injection of fibrinogen promotes inflammatory demyelination and axonal damage in the corpus callosum and spinal cord of multiple sclerosis (MS) and experimental autoimmune encephalomyelitis (EAE) mouse model, respectively, (Davalos et al., 2012; Ryu et al., 2015). It is also indicated that demyelination and white matter injury in the striatum cause motor dysfunction in many neurological diseases like Parkinson’s disease, Huntington’s Disease, and Stroke (Jeong et al., 2021; Nair et al., 2022; Yang et al., 2022). Clinical imaging shows that structural changes in the striatum correlated with motor performance deficits in MS patients (Cavallari et al., 2014). Furthermore, cuprizone-induced demyelination in the striatum causes motor dysfunction in a mouse model of MS (Mandolesi et al., 2019), indicating that striatal demyelination and axonal damage may contribute to changes in motor function. Based on previously published studies and current findings, we think the massive increase of fibrinogen extravasation in the striatum of 15-month-old AD mice induces demyelination, axonal degeneration, and finally motor dysfunction.

While we have not examined vascular cellular homeostasis in this study, one possible mechanism could be loss of pericyte coverage, as this cellular population has a well-established role in maintaining the BBB and cerebral vascularization (Winkler et al., 2014). Pericyte deficit mouse models exhibit increased BBB permeability, microvascular degeneration and expression of proinflammatory molecules (Armulik et al., 2010; Bell et al., 2010; Daneman et al., 2010; Nikolakopoulou et al., 2019). Additionally, pericyte dysfunction results in loss of myelin and axons in a manner similar to that seen in aging AD mouse models, reflecting a concurrent increase in fibrinogen extravascularization (Montagne et al., 2018). Loss of pericyte coverage is seen in AD patient brains and during AD disease progression, coinciding with BBB breakdown, microvascular degeneration and fibrinogen extravasation in AD (Sengillo et al., 2013; Halliday et al., 2016). Moreover, protection of pericyte damage improved myelination and motor function after LPC-induced demyelination in the spinal cord (Muramatsu et al., 2015) suggesting that pericyte could be a key regulator of motor function. The molecular mechanism how loss of pericyte function directly affects striatal fibrinogen extravasation and in turn motor function is not yet clear but suggests an interesting mechanism which should be assessed in future studies.

Although the hallmark symptoms of AD are loss of memory and impaired cognition, balance and motor dysfunction are also observed in AD patients and correlate with disease progression and severity (Scarmeas et al., 2004). In large scale human studies of AD patients, almost 25% showed disruptions in balance while 18% demonstrated gait disorders (Mazoteras Munoz et al., 2010; Tangen et al., 2014). In particular AD patients exhibit decreased “stability to gait,” a measure of walking balance and speed, and their performance in this assay continues to decline as disease progresses (Tangen et al., 2014). Interestingly, motor dysfunction is observed in the pre-clinical stage of AD development, suggesting that it may be a potential earlier indication of the disease (Buchman and Bennett, 2011; Montero-Odasso et al., 2014). Furthermore, poor lower limb motor function in mild cognitive impairment patients, correlates with an increased risk of AD development (Aggarwal et al., 2006). These studies provide evidence that motor dysfunction is a frequent and early deficit in AD afflicted patients.

Despite accumulating evidence that motor functions, such as gait, balance and locomotion, are greatly affected in Alzheimer’s disease patients, little is known about the underlying pathological basis of these deficits. In the present study we present a novel correlation between cerebrovascular deficits and reduced motor function in a mouse model of AD. We found that over the course of aging, this mouse model exhibits vascular degeneration, con-current accumulation of extravasated fibrin(ogen) as well as axonal demyelination and damage that is restricted in its severity to the striatum. These same mice display impaired motor function at the same age point, suggesting that this vascular dysfunction and demyelination induced axonal degeneration may contribute to the behavioral deficits observed. These findings provide possible mechanism underlying motor dysfunction in AD and suggest that vascular function and myelin architecture in the striatum may be an important target for future studies and potential treatments.

## Data availability statement

The original contributions presented in the study are included in the article/Supplementary material, further inquiries can be directed to the corresponding author.

## Ethics statement

The animal study was reviewed and approved by Rockefeller and Rutgers Institutional Animal Care and Use Committee.

## Author contributions

HB-R, AC, AR, PS, and HA performed experiments. HB-R, AR, AC, PS, and HA analyzed data and wrote the manuscript. Z-LC, and HA designed the study. AC, Z-LC, EN, SS, and HA participated in interpretation of data and manuscript preparation. All authors contributed to the article and approved the submitted version.

## Funding

This work was supported by the National Institute of Health NS104386 (HA), AG078245 (HA) and NS10668 (EN and SS), Cure Alzheimer’s Fund, Alzheimer’s Association, Rudin Family Foundation, and John A. Herrmann, Jr. SA.

## Conflict of interest

The authors declare that the research was conducted in the absence of any commercial or financial relationships that could be construed as a potential conflict of interest.

## Publisher’s note

All claims expressed in this article are solely those of the authors and do not necessarily represent those of their affiliated organizations, or those of the publisher, the editors and the reviewers. Any product that may be evaluated in this article, or claim that may be made by its manufacturer, is not guaranteed or endorsed by the publisher.
