# Supplementary material for: Striatal fibrinogen extravasation and vascular degeneration correlate with motor dysfunction in an aging mouse model of Alzheimer’s disease
Source: Front Aging Neurosci. 2023 Mar 9;15:1064178. doi: 10.3389/fnagi.2023.1064178 (PMC10034037; doi:10.3389/fnagi.2023.1064178)
Supplement: Supplementary file 1 [file Data_Sheet_1.docx]

**Supplementary Figures**


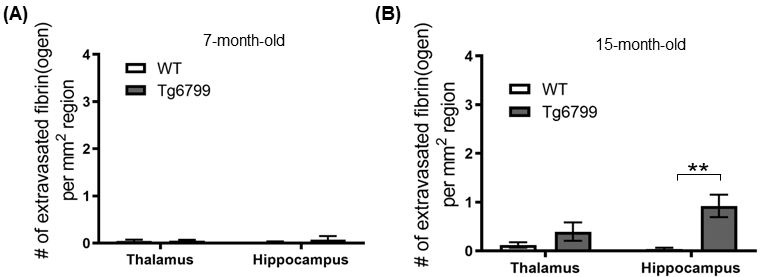


**Supplementary Figure 1.** Extravasated fibrin(ogen) levels in the thalamus and hippocampus of 7- and 15-months AD mouse model. Number of extravasated fibrinogen was measured in the thalamus and hippocampus at 7-months (A) and 15-months (B) Tg6799 AD and WT littermate mice. All numerical values presented in graphs are mean ± SEM. Statistical significance was determined using two-way ANOVA with Post-hoc pairwise t-test was applied by correction the P-values with the Bonferroni procedure **, P < 0.01; n = 5 per group).

**
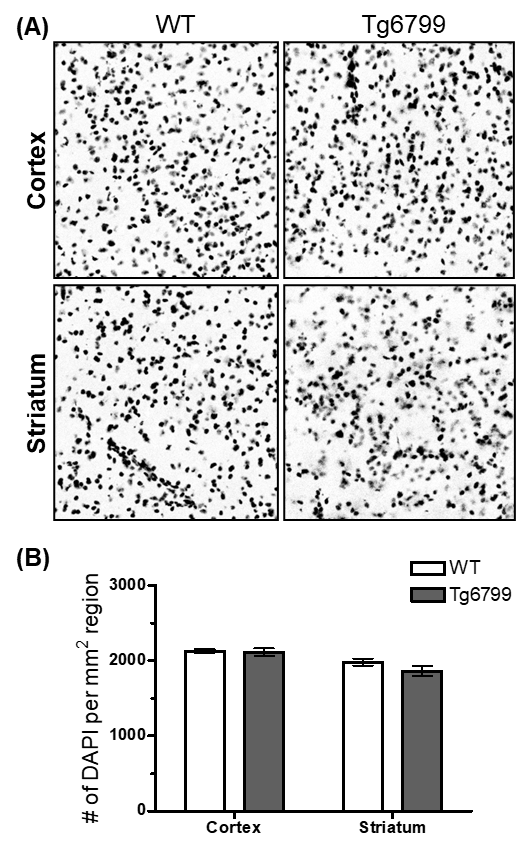
**

**Supplementary Figure 2.** Nuclear staining in the cortex and striatum of aged AD brain. (A) DAPI stained images of Tg6799 AD and WT littermate controls in cortex and striatum regions to support the immunohistochemical results performed in this study. (B) Number of DAPI stained cells were measured in cortex and striatum using Image J between Tg6799 AD and WT controls. Data was presented as mean ± SEM and analyzed by two-way ANOVA with post-hoc multiple comparison using Bonferroni procedure (n = 4 - 5 per group).


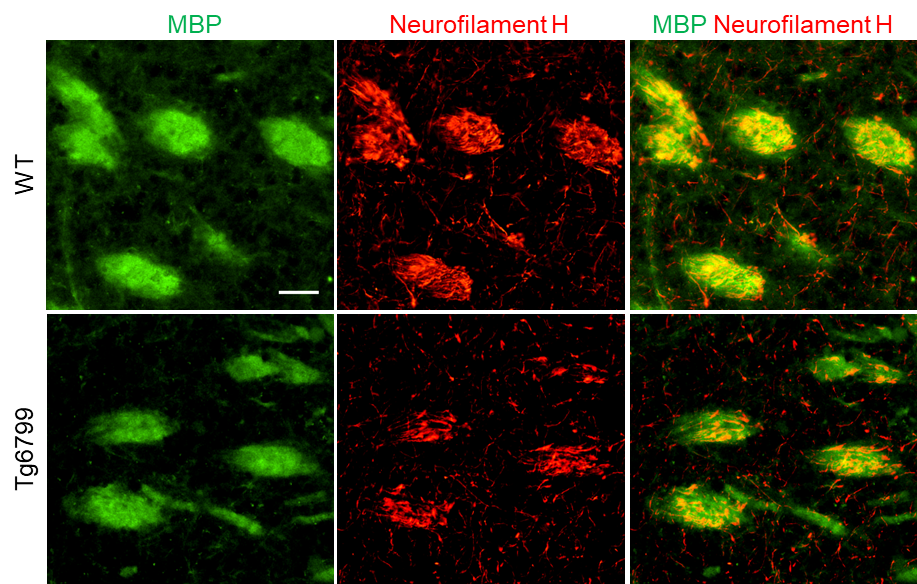


**Supplementary Figure 3.** Representative image showing Myelin Basic Protein (MBP) and neurofilament heavy (H) chain immunohistochemistry in striatum. Myelination in white matter striosome and grey matter matrix area in the striatum is visualized by MBP (green) and neurofilament H (red) staining in the WT mice which is prominently reduced in Tg6799 mice striatum (Scale bar, 50 μm).

**
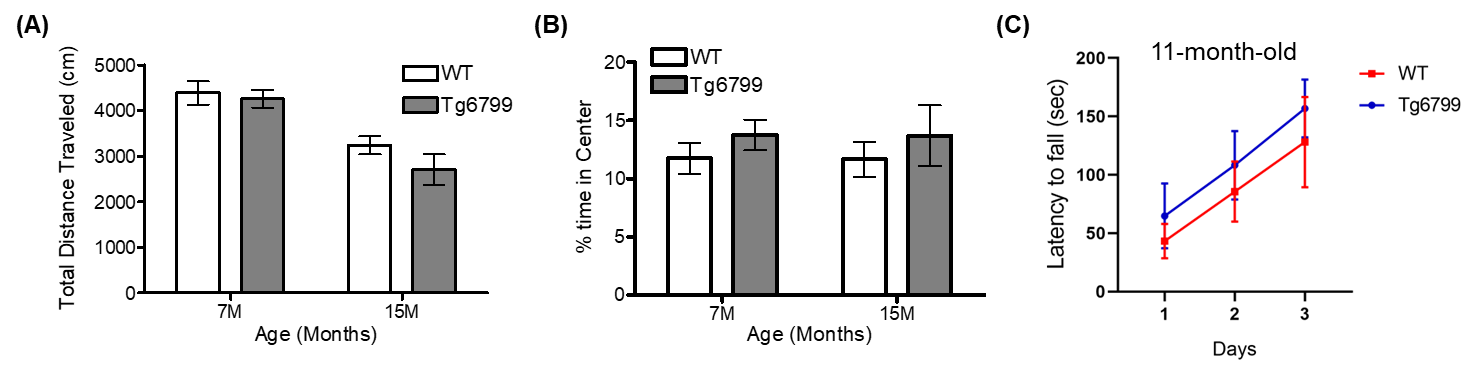
**

**Supplementary Figure 4.** Open field test measurement in AD mice. Basal level of locomotor activity and anxiety in Tg6779 AD mice and age-match WT mice at 7-months and 15-months were performed in open field test. The distance travelled (A) and the percentage of time spend in the center (B) were recorded (n = 6 - 10 per group). C) Motor dysfunction measured by Rotarod test at 11-month-old in Tg6799 and WT controls. Latency of fall from the rod was analyzed for three consecutive days (n = 6-8 per group). Data were analyzed by two-way ANOVA with post-hoc multiple comparison using Bonferroni procedure and shown as mean ± SEM.
